# Effectiveness of a grant program's efforts to promote synergy within its funded initiatives: perceptions of participants of the Southern Rural Access Program

**DOI:** 10.1186/1472-6963-8-263

**Published:** 2008-12-18

**Authors:** Donald E Pathman, Emmeline Chuang, Bryan J Weiner

**Affiliations:** 1 Cecil G. Sheps Center for Health Services Research, University of North Carolina at Chapel Hill, CB#7590, 725 Martin Luther King Blvd, Chapel Hill, NC, 27599, USA; 2 Department of Health Policy and Management, Gillings School of Public Health, University of North Carolina at Chapel Hill, McGavran-Greenberg Hall, CB# 7411, Chapel Hill, NC, 27599, USA

## Abstract

**Background:**

Foundations and public agencies commonly fund focused initiatives for individual grantees. These discrete, stand-alone initiatives can risk failure by being carried out in isolation. Fostering synergy among grantees' initiatives is one strategy proposed for promoting the success and impact of grant programs. We evaluate an explicit strategy to build synergy within the Robert Wood Johnson Foundation's Southern Rural Access Program (SRAP), which awarded grants to collaboratives within eight southeastern U.S. states to strengthen basic health care services in targeted rural counties.

**Methods:**

We interviewed 39 key participants of the SRAP, including the program director within each state and the principal subcontractors heading the program's funded initiatives that supported heath professionals' recruitment, retention and training, made loans to health care providers, and built networks among providers. Interews were recorded and transcribed. Two investigators independently coded the transcripts and a third investigator distilled the main points.

**Results:**

Participants generally perceived that the SRAP yielded more synergies than other grant programs in which they had participated and that these synergies added to the program's impact. The synergies most often noted were achieved through relationship building among grantees and with outside agencies, sharing information and know-how, sharing resources, combining efforts to yield greater capacity, joining voices to advocate for common goals, and spotting gaps in services offered and then filling these gaps. The SRAP's strategies that participants felt fostered synergy included targeting funding to culturally and geographically similar states, supporting complementary types of initiatives, promoting opportunities to network through semi-annual meetings and regular conference calls, and the advocacy efforts of the program's leadership. Participants noted that synergies were sometimes hindered by turf issues and politics and the conflicting perspectives and cultures of participating organizations and racial groups. Inadequate funding through the SRAP, restricting program involvement to only a few needy counties, and instances of over- and under-involvement by the program's leadership were sometimes felt to inhibit synergies and/or their sustainability.

**Conclusion:**

Participants of the SRAP generally perceived that the SRAP's deliberate strategies yielded synergies that added to the program's impact.

## Background

Foundations and public agencies often support society's health and social goals by creating grant programs that fund stand-alone "categorical" initiatives. Experience has shown, however, that single, narrowly-targeted initiatives operating in isolation often gain little visibility, do not have sufficient impact to achieve their programmatic goals and do not attain sufficient momentum or attract enough other support to become self-sustaining [1]. For example, consider a hypothetical federal grant program in the U.S. designed to increase the number of racial minorities in medicine by providing states with funds to award medical scholarships to minorities. This program might not be effective in yielding more minority physicians if states' initiatives do not work together to learn from one another and are not connected to local minority communities, career guidance counselors at local colleges, and financial aid staff of the region's medical schools. States' funded initiatives will also be ineffective if they do not link with programs that address other important barriers to medical careers for minorities, like inadequate secondary education opportunities.

Promoting synergy – when two or more organizations work together so their combined effect is greater than the sum of their individual effects – may be a useful approach to overcoming the common limitations of categorical grant programs. In its richest form, synergy entails more than creating relationships and exchanging information; it involves the merging of complementary perspectives, resources and skills in ways that produce new and greater value than would otherwise be achieved. The vitality, impact and sustainability of grant-funded initiatives might be strengthened if, from the outset, they are structured to make use of synergies and grantees are provided with opportunities and the means for linking with other grantees, initiatives and organizations. Synergy might help funded initiatives avoid the pitfalls of isolation.

The Robert Wood Johnson Foundation's Southern Rural Access Program (SRAP) was a grant-funding program that supported four related types of initiatives to bolster basic health care services within select rural counties in eight states in the southeastern U.S. The SRAP intentionally sought to promote synergy to increase program impact; it helped funded initiatives link with other initiatives within and across states and with other initiatives in their states.

Despite its promise, there has been little formal study of how grant programs can best foster synergies among their grantees and of whether synergy, indeed, promotes program outcomes. The goals of this evaluation were to assess the kinds of synergy that developed, and failed to develop, over the SRAP's eight years and to identify the design-features of the program that participants felt promoted synergies. This evaluation also assessed participants' perceptions of how synergies contributed to program success. Data for this evaluation were from telephone interviews with the SRAP's key participants conducted shortly after the program ended.

### Synergy

Synergy refers to cooperative interaction among groups that creates an enhanced combined effect. In business, the potential for synergy is typically conceived as the closeness or relatedness of the resources or capabilities of two enterprises, such as acquired subsidiaries or merged parts of a corporation [2-6]. Economic theory suggests that when the costs of producing outcomes separately exceed the costs of joint production, firms can achieve economies of scale or scope through the business strategies of diversification, merger, acquisition, or joint venture [7-10]. By combining resources and activities, firms can realize efficiencies from increasing the scale of production or from decreasing the scope of marketing or distribution. Drawing on this theoretical insight, researchers have examined whether firms that acquire other firms with related resources or capabilities realize greater financial returns than those that acquire other firms with unrelated resources or capabilities. Some studies have found such an effect, while others have not [6,11]. These inconsistent findings have simulated some researchers to look beyond the relatedness of firms' resources or activities (i.e., the promise or potential for synergy) and instead focus on the administrative mechanisms that firms employ to realize potential synergies [4,12-15].

In the health field, synergy has been conceived broadly as the advantages that groups and organizations realize when they collaborate through partnerships, networks, and alliances [16-20]. The pursuit of synergy is often what drives groups and organizations to form partnerships, coalitions, and other cooperative ventures to improve health [18,21-23]. Although practitioners, consultants, and experts have written a great deal about the value of synergy [19,24-28], health services researchers have only recently begun theorizing about, developing measures of, and empirically assessing synergy in collaborative partnerships to improve health [29-33]. In a landmark article, Lasker and Weiss defined synergy as "the breakthroughs in thinking and action that are produced when a collaborative process successfully combines the complementary knowledge, skills, and resources of a group of participants" [33]. Using this conceptual definition, Lasker and her colleagues developed a measure of synergy and examined its determinants in community health partnerships [34]. They found that partners' perceptions of synergy were linked to collaborative styles of partnership leadership, careful use of partners' time and resources, and, to a lesser extent, the partnership's administrative and management processes. To date, research has not examined whether community health partnerships that exhibit higher levels of synergy are more efficient, effective, influential, creative, or durable. However, Lasker and Weiss contend that partnerships that generate synergy think in new and better ways about the problems they face, take more comprehensive actions to address those problems, and develop stronger and more supportive relationships within the community [33]. These advantages can be seen as merely means to other ends, or they can also be seen as desired outcomes in their own terms.

Although much work has been done to advance knowledge of synergy in community health partnerships, this article seeks to fill two important gaps. First, the emphasis that Lasker and her colleagues place on new and better ways of thinking obscures other forms of synergy that partnerships could produce, which may be as or more valuable. Second, the emphasis they place on partnership-level determinants of synergy obscures the potentially powerful role that foundations and other funding agencies can play in promoting or inhibiting synergy in partnerships. As a condition of funding and through ongoing interactions with grantees, foundations and public agencies can influence the goals that partnerships choose, the groups and organizations that are brought to the table, the governance and administrative processes they use, and the activities they perform. Given the time, energy, and resources that foundations and public agencies invest in partnerships, a stronger knowledge base about programmatic design features that support synergy development could strengthen efforts to improve health through collaboration.

### The Southern Rural Access Program

In the mid-1990s it was well known that health indices in the rural Southeastern U.S. lagged behind other regions of the country and that poverty and lack of health insurance were more common. Shortages of physicians and other health care professionals plagued the efforts of many southern rural communities to provide health care locally.

The Robert Wood Johnson Foundation, the largest health philanthropy in the U.S., created the Southern Rural Access Program to address the chronic health care delivery problems of selected needy rural counties in eight Southeastern states – Alabama, Arkansas, Georgia, Louisiana, Mississippi, South Carolina, Texas and West Virginia [35]. The stated goals of the program were to (1) increase the supply of health care providers in underserved areas, (2) strengthen state and local health care infrastructures, and (3) build capacity at the state and community level to solve health care problems. Faculty of the Rural Health Policy Center of Penn State University College of Medicine served as the SRAP's National Program Office (NPO), managing the program and providing technical assistance to grantees.

The SRAP's work was initiated in 1997 when staffs of the Foundation and the NPO invited health care leaders within each of the eight states to submit proposals for four types of initiatives: (1) "pipeline" initiatives to prepare and guide middle school, high school and college students into health careers, (2) recruitment and/or retention initiatives to help physicians and other health professionals find work in the program's targeted rural areas and then remain there, (3) rural health network initiatives to link local health care organizations – typically hospitals and practices in adjacent counties – to promote service coordination, group purchasing, and other collaborations as they saw advantageous, and (4) revolving loan initiatives to offer low interest loans to help rural providers afford needed capital equipment. Initial three-year awards were made in 1998 to consortia in all eight states, with subsequent continuation awards made every two to three years. Funding for the program formally ended in the spring of 2006, after a total investment of $36 million. Among the findings of evaluations of various aspects of the SRAP were that (1) primary care physician numbers in the SRAP's high-poverty rural counties grew faster than in these states' other, non-targeted high-poverty rural counties [36], (2) the program's investment of $7 million to establish revolving loan programs to meet the capital needs of rural healthcare providers was leveraged many times over to expand this seed capital with funds from other foundations and public sources; by 2006 these programs had made about 100 loans totaling approximately $131 million [37,38], and (3) pipeline initiatives were generally unsuccessful due to their small size and the lack of cooperation of schools [37].

### The Planned Use of Synergy within the Southern Rural Access Program

Building synergy was a central strategy of the SRAP. The overarching planned approaches for stimulating synergy were to layer related types of initiatives within geographically bounded areas where the staffs of initiatives would naturally come in frequent contact, and to promote partnerships among the program's numerous participating organizations within and across states. A key management approach was to create many opportunities for all possible partners – local health care organizations, community leaders, state agencies, funding institutions, policy experts and policy makers – to come together around their shared goals with the expectation that synergies would emerge.

Specific features of the program's design and management intended to foster synergy included:

#### • Involving states within a specific region of the U.S

Unlike most grant programs sponsored by national philanthropies and federal agencies, only eight southeastern states were invited to participate in the SRAP: there was no wider open competition for funds. It was thought that a regional approach would help participants from states with similar cultures, policy environments, strengths and challenges develop trust, learn from one another and build cohesion.

#### • Overlaying four types of initiatives

The SRAP funded four types of related initiatives anticipating that they would be more effective together than operating individually.

#### • Focusing initiatives within a cluster of contiguous counties

As initially envisioned for the SRAP and reinforced during states' 2002 grant renewal process, each grantee targeted its initiatives to a group of 16 to 31 contiguous counties that were generally among their most socio-economically disadvantaged.

#### • Requiring joint leadership and planning

The program required grantees in each state to enlist a broad coalition of health care organizations and policy makers that was to prepare a single proposal for their state [35]. Proposals were to name a single lead agency and designate a broadly representative advisory board of state and local stakeholders. The project's work was to be subcontracted to a number of organizations within each state to foster broad program buy-in.

#### • Fostering partnerships with other funding sources

By the requirements for program participation and the encouragement of NPO staff, grantees were pushed to seek additional financial support from a variety of public and private sources and philanthropies. Among the approaches was to create the "21^st ^Century Challenge Fund" through which SRAP participants could request additional funding from the RWJF for new, innovative initiatives if they secured co-funding from local and regional philanthropies [35]. The intent was to build grantees' ties with financial institutions and other funding agencies that would become partners in promoting the expansion, impact and longevity of SRAP initiatives.

#### • Fostering program-wide communication

The NPO, with grantee co-leadership, organized twice yearly, two-day meetings of program personnel from all participating states. Meetings featured grantee-led sessions highlighting "best practices" and "lessons learned" to promote idea-sharing among the staffs of similar initiatives across the states. Meetings provided ample opportunities for socializing to build relationships and an esprit de corps. The NPO also led monthly conference calls of all grantees to address shared issues.

#### • Promoting collaboration through ongoing technical assistance

Throughout the program, the NPO helped link the SRAP's participating organizations with local and regional health care, financial and policy organizations. NPO staff served as hands-on facilitators and "cheerleaders" for collaboration.

#### • Providing new funds for between-state planning

In 2002 the grantees requested and received from the Foundation an additional $600,000 to form a new regional collaboration under their own leadership. The intent of the newly created "Southern Regional Health Consortium" was to provide a separate, grantee-directed forum within which they could further their joint work and plan for continued collaboration beyond the funded term of the SRAP.

## Methods

### Design and Data Sources

We used a qualitative interview approach to assess whether the SRAP's design indeed promoted synergies and to learn whether these synergies were seen as helpful to grantees' efforts and to program impact. We interviewed 39 key informants who were 37 SRAP participants from the eight states and two individuals from the SRAP's National Program Office. Interviewed participants included program directors from all states, leaders of the various funded initiatives who were typically subcontractors on states' grants, and front-line project personnel who were typically participating educators, loan officers, practice management consultants and recruiters. Interviews with NPO staff were used only to clarify the SRAP's structure and operations and understand the rationale behind their design. The information provided by the NPO staff was coupled with historical information from states' grant applications, program logic models [39], the papers published in a special issue of the *Journal of Rural Health *devoted to the SRAP [40], and with our experiences working as the program's evaluators over its eight-year history.

### Data Collection and Analysis

Interviews were conducted by telephone and typically lasted about 30 minutes. To promote open disclosure, confidentiality was assured and interviews were carried out by a research assistant who was new to the SRAP evaluation and not known to the interviewees. With participants' permission interviews were recorded. The research protocol was presented to, and exempted from, full review by the Committee on the Protection of the Rights of Human Subjects of the University of North Carolina's School of Medicine.

During interviews, interviewees were provided with a definition of synergy, then using a semi-structured interview guide [see Additional file 1] they were asked for specific examples of any synergies that they had observed through their SRAP involvement: participants were not prompted about specific types of synergy. Participants were also asked, without specific suggestions, about factors within or outside the program they felt enhanced or inhibited each synergy they identified.

Interview recordings were transcribed verbatim. For analysis, transcripts were segmented into non-overlapping text units of the fewest transcribed lines that retained coherent meaning assigned to various codes [41]. An initial list of codes and definitions was developed based on the literature on synergy, pilot tested with a sample of five transcripts, and then refined and expanded [42]. The remaining interviews were then independently coded by two investigators, reviewed by the third investigator, and then discussed by all three members of the research team until consensus was achieved on best coding. The list of codes was continually refined and supplemented as analysis proceeded.

Two of the investigators extracted the themes and principal points of the text segments related to each code. The third research team member then reviewed and further distilled this information to key points, which were verified by the other investigators.

## Results

Seven types of synergies were mentioned by more than one-quarter of the interviewed SRAP participants; these are described below and in Table 1. We include quotes to clarify participants' understanding and perspectives on each type of synergy and list in the text the kinds of outcomes participants attributed to these synergies. We also list the aspects of the SRAP that participants felt fostered each type of synergy.

**Table 1:** Synergies frequently noted by participants of the Southern Rural Access Program

**Synergies**	**Definitions**	**Illustrative Quotations**
Relationship Building	Creating relationships between organizations and individuals, whether at the community, state or inter-state level.	"I believe probably that there were preexisting working relationships. However, I think there were some actual friendships that grew out of this. Because of the ongoing work on the project...with folks that I didn't know before, I will pick up the phone and say "Hey, let's go to lunch and talk about some things."
Shared Information, Shared Know-How	*Shared information *is communicating substantive information ("know-what") between partnering organizations. *Shared know-how *refers to communicating procedural ("how-to") information.	"...we worked directly with the other loan funds that were developed in the other states. We shared policies and procedures, ideas, how the structure runs, what worked in some place and what worked in other places. We identified funding sources and shared those ideas with other loan fund staff in other states. During the development process it all helped us learn together how to do health care lending."
Shared Resources	Financial, human and material resources that collaborating organizations pool in order to carry out an activity or program.	"Yeah, the lead agency of the retention and recruitment component – the Primary Healthcare Association – already had a partnership with the Department of Health...but through the SRAP, oh man, it was enhanced and strengthened drastically because we were able to hire a staff recruiter. We placed that recruiter at the Department of Health, in their office, and then two years later the Department of Health, not only were they very satisfied with the partnership, they were able to contribute funds to partially support our recruiter's salary and time."
Increased Capacity	Collaborations that allowed participants to increase the volume of their activities and output	"We were able to make a lot of community partners through these programs and I saw lots of benefits in all the programs. This AHEC is hosted by the state university and we have worked with this university in the past, with the nursing students and the other students, but the SRAP gave us the opportunity to work with other colleges and universities from this region,...so it helped us expand and develop a much stronger program advising college students and directing them into health careers programs. We definitely made some good community partners with some other colleges and universities in the East Texas area."
Cumulative Impact	The additive or multiplicative effects that result from combining distinct but complementary activities and programs	"If I, as a regional recruiter, recruited a provider and they needed some special financial assistance, I could send them the information on our revolving loan fund... There were a lot of times, too, that I used practice management. There were times that I would see some improvements that could be made in different primary care practices and I could refer them to our practice management consultant."
Shared Voice	Efforts among partnering organizations to come together to vie for greater political or market power	"We were also able, over the course of time, to develop with the University of Mississippi Medical Center a Scholarship-Loan Forgiveness program for individuals who would go on to be accepted to the Ole Miss Medical School and would agree to come back to the rural community and become family practice physicians. The legislature actually assisted with the development of funding for that program. There was a lot of people who ordinarily wouldn't lobby together, down at the capital, speaking with one voice."
Gap Spotting and Closure	When organizations recognize needed services or programs missing locally and then provide these services to complement or enhance existing programs	"So there was an observation from the loan fund side that practice management was an activity where clearly professional help was needed. It was being provided in the market to some who can afford, but others who couldn't weren't gaining access to it. It was strongly suggested also by [the NPO] and, as I understand it, by [the state project director] to fund that element. Given the fact that it was encouraged and the need was observed as being there, that led to the generation of it."

### Synergies Frequently Noted by SRAP Participants (Table 1)

#### Relationship Building

When asked about synergies they experienced in the SRAP, virtually every interviewed participant mentioned that they had formed new relationships and strengthened existing ones with organizations and people within their states. Participants expressed positive feelings of connectedness and camaraderie and indicated that relationships provided useful partners and sources of information and resources. Several participants noted that the within-state relationships they formed through the SRAP enabled their programs to expand capacity and reach new audiences. Others noted that within-state relationships allowed them to share perspectives and resources, identify gaps in existing programs, and find common ground from which to work together. Some participants indicated that the friendships formed within the SRAP were the most important benefits of their involvement in the program and might prove to be the most enduring.

"I just hated to see the project come to an end because I think we had done so much good work. Some of that work is going to continue and some of it is not. But again, I think the relationships that have been formed are there and that at least from that standpoint, those things will continue to go forward. If nothing else, I now know the lady that runs the community health center at Hickory Flat and I can call her if I need her. She can call me."

Participants felt that within-state relationships were fostered from the very outset of the SRAP when all possible stakeholders were invited to the introductory meetings held within each state. They felt that relationships were promoted by the program's use of overlapping initiatives, the coordination provided by lead agencies, and by states' initiatives being subcontracted to a variety of organizations that then worked together.

"Actually, the whole heart of the SRAP when the grant was announced created a lot of synergies in the communities in that there were a group of somewhere between 200 to 250 people that convened."

Participants also frequently remarked on the relationships they established with people and organizations in *other *SRAP states. These relationships often started with information exchanges at the semi-annual grantee meetings and from introductions made by the NPO. Cross-state relationships were promoted by the comfort and commonality participants felt with people involved in similar initiatives in states very much like their own. Participants often noted that cross-state relationships involved sharing information and know-how and sometimes led to site visits across states.

#### Shared Information and Know-How

Sharing information and know-how was mentioned by participants who were involved in all four types of initiatives and was noted to occur between organizations located within the same state and organizations in different states. *Shared information *included communications about the issues and opportunities within a field and talking about partners' activities, experiences and "best practices". *Shared know-how *primarily involved sharing practical knowledge and providing technical assistance to one another.

Participants frequently noted the importance of the program's semi-annual grantee meetings in allowing organizations in different states to share information and know-how. The NPO's role as a facilitator of exchanges was also cited. Several grantees noted that the program's regional focus in the Southeast fostered acceptance and trust among grantees, making it easier for organizations across states to learn from one another.

"The fact that it was Southern states, when you meet, you have such similar issues in your home state that the ideas and suggestions you could share were useful and beneficial. I've been in a lot of other grant situations where you bring in folks from around the country, and the circumstances in each state are so unique,... what you can pull off in Michigan and Oregon, well, that dog don't hunt in South Carolina."

#### Shared Resources

Many SRAP participants identified instances where their organizations shared resources with other SRAP-participating and non-participating organizations. Participants provided examples of sharing services (e.g., partners distributing marketing materials for one another without charge), human resources (e.g., partners sharing the costs of salaried positions), support services (e.g., providing free office space to a partner), and funding (e.g., revolving loan programs that partnered with federal, state and foundation sources to build their capital funds). Participants remarked on the important roles played by the NPO and states' lead agencies in helping SRAP participants meet new collaborators who could become sharing partners.

#### Increased Capacity

Beyond the many instances where participants noted that funding from the SRAP directly allowed them to expand the size of previously existing initiatives – which is not synergy per se – some participants noted that their collaborations with other SRAP participants also led to greater program output, which is synergy. Especially common were examples of synergies from partnering with new funding agencies, particularly in building revolving loan fund capital.

"We developed partnerships with a number of traditional lenders,... [A bank] helped us with the underwriting and the servicing of the loans. They helped us pretty much set up the little details of the program, plus they furnished some of the funding for the projects that we worked on, so they were a key partnership. We also developed a partnership with the Rural Development arm of the USDA. We were able to get two grants from them; one was a direct grant for $900,000 and one was an Intermediary Relending Program grant for $500,000."

Interviewees felt that the SRAP's semi-annual all-grantee meetings, monthly conference calls and NPO guidance all helped participants identify and learn how to work with new funding partners.

#### Cumulative Impact

Participants noted various ways in which the availability of the complementary services of the SRAP's several initiatives augmented the impact of each. This happened most often when the staff of one initiative referred clients – health professions students, physicians and medical practices – to another SRAP initiative. Cross-initiative referrals augmented the visibility of initiatives and promoted the use of their services. As intended in the program's design, participants felt that specific local health care providers sometimes benefited from the services of more than one initiative.

"So, I guess the combination between practice management, locum tenens, and recruitment and retention all helped strengthen the rural physician base in the community."

#### Shared Voice

About one-quarter of participants cited synergies that stemmed from combining the voices and power of participating organizations to gain greater recognition from legislators and funding agencies. This happened both within and between states. Instances of shared voice were not attributed to specific aspects of the SRAP's design but were more loosely seen as emerging from people and organizations with shared goals coming together through the program.

"The political influence has probably been the most important... this group has provided a cohesive rural voice that has always said... we are speaking for rural Alabama and we say, "You ought to do this", and that helped."

#### Gap Spotting and Closure

About one-third of participants noted instances where interactions between SRAP organizations, or between SRAP-supported organizations and other organizations in their states, led to the identification and then filling of gaps in services within communities. Gap spotting and closure were fostered by the SRAP's approach of funding several initiatives addressing the same or related health care problems within geographically circumscribed areas and by the assistance that NPO staff provided to grantees.

### Synergies Mentioned Infrequently Within the SRAP

Fewer than 5 to 10 of the 39 SRAP participants we interviewed mentioned some of the types of synergies that figure prominently in the corporate literature on synergy. We heard only a few mentions that collaborations allowed participants to *avoid duplicating services*, that is to recognize and reduce redundant and wasteful capacity when two organizations are offering similar programs. Also, only two participants gave examples of benefiting from *economies of scale*, where program unit costs of production are reduced by two organizations coming together to jointly produce a larger volume of services. We suspect that these two types of synergy were seldom realized because the SRAP's targeted counties were profoundly poor and their resources scarce, so there were few redundant services that needed to be scaled back. Also, the SRAP's initiatives generally provided personally delivered services, which less often lend themselves to economies of scale.

Another type of synergy popular in business – *creating spin-off initiatives and organizations *from parent collaborating organizations – was reported from only two kinds of collaborations within the SRAP. The first was the creation of the Southern Regional Health Consortium (SRHC), which emerged from the relationships that formed among the lead agencies of the eight states. The second was the handful of instances where relationships that formed with state governmental agencies eventually led these agencies to assume the ongoing funding and, in some cases, administrative control of the initiatives as the SRAP's funding ended.

### Barriers to Synergy

Most participants could readily identify specific barriers to synergy, which were principally of three types (Table 2):

**Table 2:** Perceived barriers to synergies

**Barriers**	**Illustrative Quotations**
*Factors Outside the SRAP*	
Turf issues and politics	"Health care in [our state] is pretty 'turfy.' When we became the lead agency... it changed a lot of interpersonal dynamics. It created huge jealousies in the state and caused many problems."
	"I guess, I felt like there were more opportunities that could have been pursued with the Office of XX... actually what happened was they sort of sat on it and didn't really do anything with it and sort of said it would be threatening to certain people. And it was probably the turf issues."
	"Politics and egos."
Differences among organizations' types, structures and agendas	"The one dynamic that probably interfered with synergy a little bit is the nature of our different work environments. I come from a private not-for-profit and [our] issues are very different... than those programs based in academia or health departments of state governments. I struggle with very different issues and sometimes there was really a lack of understanding about the different environments we each come from."
	"I think some factors [that kept synergies from developing] are in that the infrastructure at some of these health professional schools generally didn't have the same type of vision for moving in that direction as far as entrance into the schools [for minorities].
Racial and cultural differences in perspectives; racial tensions	"The other thing that we noticed that was very unfortunate is that these [coalitions] tend to form along racial lines. There will be a black health care coalition and a white health care coalition, and they won't talk to one another. We, even though we tried, were never successfully able to get those folks to break bread together. That was a failure that was disappointing."
*Features of the SRAP*	
NPO was sometimes over- involved or under-involved in managing collaborations	"Something that just made it worse was national program staff inserting themselves in the middle of it when they didn't really have a full understanding of the dynamics."
	"I think the SRAP program office could have intervened in states where they knew things were not going well and said, 'You either take this and this and do with it as we say because we have the money. You need to do it, or you need to find somebody else to lead the program."'
Too few program dollars to promote or maintain synergies	"The folks that early on I was engaged with, for the most part, stayed involved to the end of the project. [But] towards the end of the project, it's fair to say as folks weren't receiving funding, some would miss a meeting here or there, and that just happens. You have to go on to other projects or go on to other things that take your time."
Limiting program involvement to only some counties within states	"You know, Robert Wood Johnson made the decision in Phase II to concentrate their efforts. I'm not sure that really proved to be as beneficial as people thought it would be. In other words, the layering on of different services in an area was not as beneficial as it could have been or as they anticipated it would be."
	"It was almost like a betrayal later on when I went to a meeting of the [my state] Southern Rural Access and was told that the decision was made to drop everyone that wasn't in the Delta. We were dropped!... I guess that's my point that I want to stress to folks is you almost hang people out to dry."
	"Well, one of our problems is in Texas we were not a statewide project. We really tried hard to bring some of the programs to the statewide level and to get the support from state agencies, and things were a challenge because we were regional."

*1. Turf issues and politics*, which arose from concerns over who would "get credit" for the successes of collaborative efforts.

*2. Differences in organizational type, structure and agendas *between collaborating organizations, which led partners to view the world differently and make them responsive to different incentives.

*3. Racial differences in perspectives and racial tensions*, which inhibited communications, trust and the functioning of some groups.

A number of factors about the SRAP itself were also noted to sometimes inhibit synergies (Table 2):

1. A few participants cited instances when the NPO needed to have been more actively involved to push collaborations forward, and others cited instances when they felt the NPO was over-involved and attempting to micro-manage collaborations.

2. Some participants felt that the program sometimes provided too few dollar incentives to support collaboration, which reduced the motivation of partners to work together. Similarly, participants noted that some previously successful collaborations weakened towards the end of the program as funding was weaned.

3. Several participants felt that geographically focusing the SRAP's initiatives on specific contiguous counties within each state was, on balance, counterproductive because it introduced a sense of exclusion, caused resentment in non-included counties within some states which hampered the program's external collaborations, and created problems for some state agencies and organizations otherwise responsible for statewide programs.

### Comparing the SRAP's Synergies to Those of Other Grant Programs

Participants were asked how the synergies they saw within the SRAP compared to what they have experienced through other foundation and public grant programs. Most had participated in other grant programs and felt the SRAP yielded more and more effective synergies. Individuals attributed the SRAP's success with synergies to each of its features intended to foster them, particularly its restriction to states within only a specific region, its requirement for joint leadership and planning, and the effectiveness of its grantee meetings.

"I think the synergies in the SRAP were very evident across state lines and across organization lines. It was much more than what I've seen with other types of programs."

### Limitations

This study assessed synergy within the SRAP only as viewed by individuals who directly participated in the program; individuals outside the SRAP who interacted with its participants and initiatives might have perceived things differently. Also, we cannot confirm participants' perceptions that synergies enhanced the reach of the SRAP. As in any study that relies on subjects' reports, social desirability might have prompted this study's participants to portray their experiences and opinions in an overly positive light and withhold negative views. It seemed, however, that subjects were quite willing to share their full opinions, both positive and negative; only a few seemed to pull their punches.

Events and factors that stand out prominently and are directly relevant to people are more likely to come to mind and be volunteered when they are interviewed with open-ended queries. SRAP participants might have overlooked some types of synergies that were less salient to them. Therefore, for example, if economies of scale were not important in the thinking of SRAP participants, then this may explain why we heard little about them in the interviews. We might have learned of more instances of economies of scale had we explicitly asked for examples of this type of synergy. Further, we might have missed some types of synergies that were difficult for subjects to describe or difficult for us, in coding, to discern from other, more commonly reported types of synergy.

## Discussion and Conclusion

According to these reports from key participants of the Southern Rural Access Program, synergy was a broadly recognized feature of the program. Participants knew the SRAP was designed to foster synergy. Virtually all participants could name one or more examples of synergy they saw grow out of their states' involvement in the program and nearly all could identify features of the SRAP's design they felt promoted these synergies. As a whole, participants felt that the SRAP yielded more and richer synergies than other grant-funded initiatives in which they had participated and they felt that synergy contributed to the program's successes.

These findings for the SRAP do not mean that synergy should be promoted in all grant-funded programs. Synergy is not always needed or appropriate. If an organization can do something well on its own, forcing synergy can be counterproductive [13]. Promoting synergy as a strategy is probably most appropriate for grant programs that address complex social problems with many underlying causes where a number of complementary initiatives are required necessitating the involvement of a number of organizations offering different capacities.

Synergy is also not always feasible [43]. Some SRAP participants we interviewed mentioned instances where efforts to promote synergy proved distracting and strained interpersonal and inter-organizational relationships. Known barriers to synergy include a lack of willing or available organizational partners, differences in organizations' disciplinary and cultural perspectives, legal or regulatory restrictions on collaboration, and disincentives built into funding mechanisms and payment systems. Because synergy is not cost and risk-free, the decision to promote synergy within programs should be instrumentally rather than ideologically driven. Are the circumstances right? Are potential partners compatible? Are program participants open to collaboration and looking for synergy? Is the program able to help grantees who don't initially value synergy learn to do so, and is it able to provide grantees with the skills they need?

Observations from the SRAP's participants suggest that if foundations and other agencies intend to promote synergy among the initiatives they fund, then it should be built into the fabric of the program and pursued through multiple strategies: synergy doesn't just happen. Able program leadership is also key. Many of the SRAP's successful synergies came about because the National Program Office staff adeptly spotted opportunities and resources to support synergy, brokered relationships, and more generally helped grantees understand, value and learn how to create synergies.

Finally, promoting synergy in grant programs may require investments in education and training to enhance grantees' collaborative competence. As Liedtka observed:

Collaboration calls upon skills that have been rarely rewarded in most organizations – listening with an open mind to the proposals of others versus selling one's own solutions harder; acknowledging and using conflict productively versus suppressing and ignoring it; leading by supporting and facilitating versus managing through authority and fiat; and designing new end-to-end value systems rather than tinkering with incremental fixes to current processes. [44]

Fortunately, the skills needed to create synergies can be acquired and sharpened. Investing in education and training of grantees, typically through technical assistance, can enhance their ability to collaborate and not only increase the level and effectiveness of synergy within a grant program, but also create an enduring community capability. The relationships formed within the SRAP and the enhanced capacity of its participants to collaborate and find synergies may be the program's most important and lasting legacy. These strengthened capacities have placed the SRAP's states and rural counties in a better position to solve their health care problems, one of the program's original goals.

## Competing interests

The authors declare that they have no competing interests.

## Authors' contributions

DEP conceived of the study, contributed to its design and coordination, participated in data coding and helped draft the manuscript. EC assisted in the design and refinement of the interview guide, recruited study subjects, conducted the study's interviews, participated in data coding and helped draft the manuscript. BJW assisted in the design of the study and its coordination, participated in data coding, and helped draft the manuscript. All authors read and approved the final manuscript.

## Pre-publication history

The pre-publication history for this paper can be accessed here:



## Supplementary Material

Additional file 1 Interview guide for SRAP synergy assessment. This guide was used by EC during phone interviews to standardize the questions posed to study participants. Click here for file
