# Supplementary material for: Effectiveness of a grant program's efforts to promote synergy within its funded initiatives: perceptions of participants of the Southern Rural Access Program
Source: BMC Health Serv Res. 2008 Dec 18;8:263. doi: 10.1186/1472-6963-8-263 (PMC2621197; doi:10.1186/1472-6963-8-263)
Supplement: Additional file 1 — Interview guide for SRAP synergy assessment. This guide was used by EC during phone interviews to standardize the questions posed to study participants. [file 1472-6963-8-263-S1.doc]

**Additional File**

**Interview guide for SRAP synergy assessment**

My name is [XX] and I work with Drs. [XX] and [XX] on the evaluation of the Southern Rural Access Program. Thank you for agreeing to talk with me today.

Before we begin, I’d like to remind you that the interview is being recorded so that Dr. [XX], Dr. [XX} and I can independently listen to and understand what participants say. Everything you tell me today will be held confidential, meaning that only members of the evaluation team will hear the tape or see its transcript. The report we create from this aspect of the evaluation will not attribute any statements about specific people, organizations or programs to specific participants. We hope that you will speak freely about your experiences with SRAP.

As you probably know, I am new to the SRAP evaluation. I am a doctoral student in the University of North Carolina’s School of Public Health. Although I have read some about your state’s SRAP activities, I haven’t worked with the people and initiatives of the SRAP and I haven’t formed any personal opinions about the program’s success. That is why I was asked to conduct these interviews.

Before we begin, do you have any questions about the interview?

Let me start by providing a definition of synergy as we are using it in this evaluation.

**Definition of Synergy**

Partnerships create synergy when participating organizations combine activities, information, resources, people and skills in ways that produce outcomes that no single organization could produce alone. In a nutshell, synergy refers to the 2 + 2 = 5 effect: more is accomplished by working together than could be achieved alone.

We are especially interested in learning about synergies that did or did not develop during the course of the SRAP. As you answer the questions that follow, please indicate as specifically as you can how the SRAP directly or indirectly contributed to any synergies that you describe.

**Questions Posed**

**1. *What was your role in the Southern Rural Access Program (SRAP)?***

**2. *Did you observe any synergy develop through the course of the SRAP or because of it?***

Prompt: for those who can’t think of any synergies, ask about synergies listed by the NPO or by previous interviewees in the same state.

|  | *Follow-Up Questions* | *Notes to Interviewers* |
| --- | --- | --- |
|  | - Could you provide an example? - What factors contributed to the synergy you just described? - What other factors contributed to the synergy you just described? - Can you point to specific aspects of the SRAP itself that promoted this synergy? - Do you think the synergy you just described would have happened anyway in your state(s) even in the absence of the SRAP? - Why do you think the synergy had not developed before the SRAP? - What outcomes resulted from the synergy you just described? - Do you think the outcomes you described would have resulted even in the absence of SRAP? | - Get a detailed description of the reported synergy including the context it which it developed, who was involved, when in the six-year course of the SRAP it occurred, how it developed and what outcomes resulted - Probe for specifics about how the SRAP directly or indirectly contributed to the synergy - Ask for more than one synergy, perhaps across different programs |

**3. *Were there other synergies you observed during the course of the SRAP or because of it?***

**[Ask the same follow-up questions as above]**

**4.** For respondents who only named synergies of a certain type (e.g., all within their state), prompt for different types/levels of synergy: Ask variations of ***“You have mentioned synergies that happened within your state. Were there also synergies that happened across states because of their participation in the SRAP?”*** or ***“You have mentioned synergies that happened for the rural recruitment program. Were there also synergies that happened for other SRAP initiatives within your state?”***

**[Ask the same follow-up questions as above]**

**5. *SRAP’s participating organizations are involved in a variety of other programs outside the grant. Did you observe any synergy develop within your state among SRAP partners and other organizations or individuals not officially part of SRAP?***

**[Ask the same follow-up questions as above]**

**6. *Can you think of any synergies that could have developed as a result of SRAP, but didn’t? I am thinking about collaborations that were suggested or attempted but didn’t succeed, or partnerships that didn’t work or didn’t last.***

|  | *Follow-Up Questions* |  |
| --- | --- | --- |
|  | - Would you offer an example or two of potential synergies that did not materialize? - What factors kept that/each synergy from developing? - What other factors kept that/each synergy from developing? - Is there anything that the SRAP could have done to address those factors? |  |

**7. *I would like to switch now to talk about your involvement in funded programs other than the SRAP. Throughout your career, have you been involved with other major grant-funded programs to improve health care or access to care for rural or poor populations, or involved in other health or social-service programs and positions?***

|  | *Follow-Up Questions* |  |
| --- | --- | --- |
|  | - Would you offer some examples of such programs? - Were any of the programs you described funded by foundations? If so, which? - How did the amount or type of synergy that developed through the SRAP compare to the amount or type of synergy from those other programs? - What accounts for the similarities or differences in the amount and types of synergy? |  |

**8. *Is there anything I didn’t ask related to synergy that you think is important, or that you would like to add?***
